# Actomyosin regulation by Eph receptor signaling couples boundary cell formation to border sharpness

**DOI:** 10.7554/eLife.49696

**Published:** 2019-09-10

**Authors:** Jordi Cayuso, Qiling Xu, Megan Addison, David G Wilkinson

**Affiliations:** The Francis Crick InstituteLondonUnited Kingdom; California Institute of TechnologyUnited States; California Institute of TechnologyUnited States

**Keywords:** hindbrain, Eph receptor, border sharpening, cell specification, actomyosin, tension, Zebrafish

## Abstract

The segregation of cells with distinct regional identity underlies formation of a sharp border, which in some tissues serves to organise a boundary signaling centre. It is unclear whether or how border sharpness is coordinated with induction of boundary-specific gene expression. We show that forward signaling of EphA4 is required for border sharpening and induction of boundary cells in the zebrafish hindbrain, which we find both require kinase-dependent signaling, with a lesser input of PDZ domain-dependent signaling. We find that boundary-specific gene expression is regulated by myosin II phosphorylation, which increases actomyosin contraction downstream of EphA4 signaling. Myosin phosphorylation leads to nuclear translocation of Taz, which together with Tead1a is required for boundary marker expression. Since actomyosin contraction maintains sharp borders, there is direct coupling of border sharpness to boundary cell induction that ensures correct organisation of signaling centres.

## Introduction

During embryo development, sharp borders form at the interface of adjacent tissues and between domains within tissues that have a different regional identity. These borders are generated by cell segregation mechanisms that establish and maintain a precise organisation of tissues (Batlle and Wilkinson, 2012; Dahmann et al., 2011; Fagotto, 2014). At some borders, a distinct boundary cell population is induced which serves as a signaling centre that regulates the patterning of cell differentiation within the tissue. The formation of a sharp and straight border enables such boundary signaling cells to be correctly organised (Dahmann and Basler, 1999). It remains unclear whether or how the induction of a signaling centre is coordinated with border sharpening. In principle, border sharpening and formation of boundary signaling cells may involve parallel mechanisms that are not directly linked. However, studies of the vertebrate hindbrain found that Eph receptor and ephrin signaling is required both for border sharpening and the formation of boundary cells (Cooke et al., 2005; Terriente et al., 2012; Xu et al., 1995), raising the possibility that there is a mechanistic link.

Eph receptor and ephrin signaling has major role in cell segregation and border sharpening in many tissues in vertebrates (Batlle and Wilkinson, 2012; Cayuso et al., 2015; Fagotto et al., 2014). Eph receptors comprise a large family of receptor tyrosine kinases that are activated upon binding to their membrane-bound ephrin ligands. Members of the EphA subclass bind to the GPI-anchored ephrinA ligands, whereas EphB receptors bind to transmembrane ephrinB ligands; an exception is EphA4 which binds to ephrinA and specific ephrinB family members (Gale et al., 1996). Upon interacting through cell-cell contact, Eph receptor and ephrin proteins are clustered and this activates signal transduction through both components, termed forward and reverse signaling, respectively (Klein, 2012; Pasquale, 2008). For Eph receptors, this involves kinase-dependent signaling that activates multiple intracellular pathways. In addition, signaling is mediated by a motif at the C-terminus of Eph receptors that binds to PDZ domain proteins. In the case of ephrinB proteins, signaling occurs through phosphorylation of conserved tyrosine residues by cytoplasmic kinases, and also through interaction of PDZ domain proteins.

Eph receptors and ephrins that have a high affinity for each other are expressed in complementary domains in many tissues (Cayuso et al., 2015; Gale et al., 1996; Rohani et al., 2014), such that activation of forward and reverse signaling occurs at the interface. Eph receptor and ephrin signaling can drive cell segregation and border sharpening through multiple mechanisms that likely depend upon whether the tissue is epithelial or mesenchymal: by decreasing cell-cell adhesion (Fagotto et al., 2013; Solanas et al., 2011), by increasing cortical tension (Calzolari et al., 2014; Canty et al., 2017), or by triggering cell repulsion (Poliakov et al., 2008; Rohani et al., 2011; Taylor et al., 2017; Wu et al., 2019). In addition, Eph-ephrin signaling has been found to regulate cell differentiation in a number of tissues (reviewed by Laussu et al., 2014; Wilkinson, 2014). The regulation of cell differentiation and segregation may be distinct and context-dependent functions. Alternatively, Eph-ephrin signaling could couple cell specification to maintenance of their organisation. For most tissues, it is unclear whether such coupling occurs, but a potential example is the formation of boundaries in the vertebrate hindbrain.

The hindbrain is subdivided into segments, termed rhombomeres (r1-r7), each with a distinct anteroposterior identity and demarcated by borders across which cell intermingling is restricted (Fraser et al., 1990). These borders are initially fuzzy and then sharpened through the regulation of cell identity (Addison et al., 2018; Wang et al., 2017) in combination with cell segregation driven by Eph-ephrin signaling (Cooke et al., 2005; Irving et al., 1996; Kemp et al., 2009; Nieto et al., 1992; Xu et al., 1995; Xu et al., 1999). Boundary cells are induced to form at segment borders (Guthrie and Lumsden, 1991) and express specific molecular markers that distinguish them from non-boundary cells (Cheng et al., 2004; Cooke et al., 2005; Heyman et al., 1995; Xu et al., 1995). In zebrafish, these include the Notch modulator, *rfng*, which by inhibiting neurogenesis promotes the maintenance of boundary cells (Cheng et al., 2004). Boundary cells are a source of neuronal progenitor cells in chick (Peretz et al., 2016), and express a number of signaling molecules that may regulate local patterning (Amoyel et al., 2005; Gerety and Wilkinson, 2011; Riley et al., 2004; Sela-Donenfeld et al., 2009; Weisinger et al., 2012). In the zebrafish hindbrain, boundary cells organise spatially-restricted neurogenesis within segments by expressing Semaphorin chemorepellants that position fgf20-expressing neurons in each segment centre (Gonzalez-Quevedo et al., 2010; Terriente et al., 2012). Clues to how boundary cells are induced at segment borders have come from studies of Eph receptor and ephrin function. Several Eph receptors are segmentally expressed in the hindbrain, in a complementary pattern to ephrinBs that they have high affinity for: *ephA4* in r3 and r5 is complementary to *ephrinB3* in r2, r4 and r6; *ephB4* in r2, r5 and r6 is complementary to *ephrinB2* in r1, r4 and r7. Disruption of Eph receptor or ephrin function leads to a decrease both in the sharpening of segment borders and in the expression of boundary markers (Cooke et al., 2005; Sela-Donenfeld et al., 2009; Terriente et al., 2012; Xu et al., 1995). These findings raise the questions of how Eph-ephrin signaling leads to boundary cell formation and whether this involves distinct pathways from border sharpening.

We set out to dissect mechanisms of signaling that underlie border sharpening and boundary cell specification in the zebrafish hindbrain. EphA4 and ephrinB3 act as a signaling pair since knockdown of either component disrupts the same segment boundaries (Terriente et al., 2012). We find that boundary cell markers are expressed in *epha4*-expressing cells and are up-regulated by forward signaling. By creating a series of truncation and point mutants in *epha4*, we show that kinase-dependent and PDZ domain-dependent signaling both contribute to regulation of border sharpening and boundary-specific gene expression. We find that boundary marker expression is regulated by myosin II phosphorylation that occurs downstream of EphA4 activation and increases mechanical tension at segment borders. Mechanotransduction that induces boundary marker expression is mediated by nuclear translocation of Taz. The regulation of actomysosin contraction by Eph signaling thus couples the maintenance of sharp borders and induction of a boundary signaling centre.

## Results

### Boundary marker expression occurs in ephA4-expressing cells

**Figure 1.**
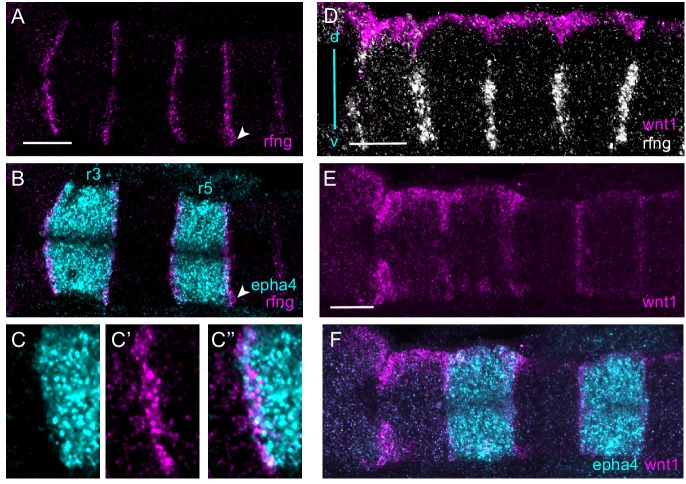
Boundary markers are expressed in epha4-expressing rhombomeres. (**A–C**) HCR stainings for *rfng* and *epha4. rfng* is mainly expressed in *epha4*-expressing cells in rhombomeres r3 and r5, with a few *rfng-*expressing cells in adjacent segments, for example r6 (arrowhead). (**C–C’’**) show a higher magnification view of an r2/r3 border. (**D–F**) Boundary expression of *wnt1* is dorsal to *rfng* (**D**) and is co-expressed with epha4 (**E, F**). (**A–C, E, F**) are dorsal views, (**D**) is a lateral view, dorsal (**d**) top, ventral (**v**) bottom. Anterior to the left in all panels. Scale bar: 50 μm.

Since *epha4* (*epha4a*) is expressed in r3 and r5 (Xu et al., 1995), and *ephrinb3* (*efnb3b*) in r2, r4 and r6 (Chan et al., 2001), this Eph-ephrin pair interacts at all borders of r3 and r5. Due to the bidirectionality of activation, knockdown of either component will lead to loss of both Eph and ephrin activation, and it is therefore not possible to deduce whether forward and/or reverse signaling regulates boundary marker expression. A clue can come from determining whether boundary cells form in *epha4*-expressing cells, *ephrinb3*-expressing cells, or both. To address this, we carried out in situ analysis using the hybridisation chain reaction (HCR) which enables sensitive fluorescent detection of multiple transcripts (Choi et al., 2016). We found that *rfng* expression which marks hindbrain boundary cells occurs in *epha4*-expressing cells at the borders of r3 and r5 (Figure 1A,B,C–C’’). *rfng* expression is also detected in a few cells that are not expressing *epha4*, which are most consistently found at the lateral edge of the r5/r6 border (arrow in Figure 1A,B). We also analysed expression of *wnt1*, which is expressed in the roof plate and in the dorsal part of hindbrain boundaries (Figure 1D). We found that *wnt1* expression in boundaries also occurs predominantly in *epha4*-expressing cells (Figure 1E,F). Boundary cell formation thus occurs in cells in which forward signaling is occurring. However, *rfng* expression also occurs in some cells that are not expressing *epha4*, which could reflect a role of reverse signaling, or a dynamic relationship between *epha4* and *rfng* gene expression.

### Border sharpening and boundary marker expression require forward signaling

**Figure 2.**
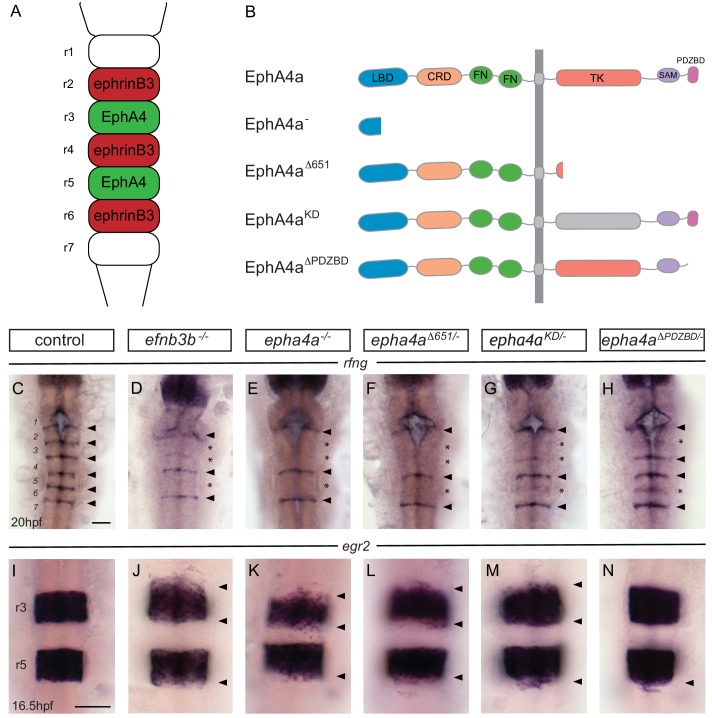
EphA4 forward signalling regulates boundary marker expression and cell segregation. (**A**) Schematic representation of the segmented expression of EphA4 and ephrinB3 in the hindbrain. (**B**) Schematic representation of the different mutant alleles of *ephA4a* generated for this study. The null allele contains an early truncation in the ligand binding domain. The *epha4^Δ651^* allele lacks most of the cytosolic domain. The *ephA4^KD^* allele contains a point mutation of a critical lysine in the tyrosine kinase domain. The *ephA4^ΔPDZBD^* mutation consists of a C-terminal truncation that deletes the PDZ-binding domain. LBD – ligand binding domain; CRD – cysteine rich domain; FN – fibronectin repeat; TK – tyrosine kinase domain; SAM – sterile alpha motif; PDZBD – PDZ binding domain. (**C–H**) *rfng* is expressed at boundaries in control embryos (arrowheads) (**C**), but is reduced or absent (asterisk) at specific boundaries in *ephrinb3^-/-^* (**D**), *epha4^-/-^* (**E**), *epha4^Δ651^* (**F**), *epha4^KD^* (**G**) and *epha4^ΔPDZBD^* (**H**) mutants. Numbers analysed for C-G are in Figure 4 legend. For H, 8/8 have decrease at r2/r3, 4/8 at r5/r6. (**I–N**) *egr2* expression in r3 and r5 has sharp borders in control embryos (I, 13/13); border sharpening defects (arrowheads) are observed in *ephrinb3^-/-^* (J; 12/12), *epha4^-/-^* (K; 17/17), *epha4^Δ651^* (L; 8/8), *epha4^KD^* (M; 6/6) and *epha4^ΔPDZBD^* (N; 2/7 at r2/r3; 7/7 at r5/r6) mutants. Dorsal views, anterior to the top in all panels. Scale bar: 50 μm.

Knockdown of *epha4* or *ephrinb3* leads to loss or decrease in expression of boundary cell markers at three borders where they interact (Figure 2A): r2/r3, r3/r4 and r5/r6 (Terriente et al., 2012); there is potential functional redundancy with *ephb4* and *ephrinb2* at the r4/r5 border (Chan et al., 2001; Cooke et al., 2001; Cooke et al., 2005). To test roles of different aspects of EphA4 signaling, we used CRISPR/Cas9 genome modification to create a series of zebrafish lines with point or truncation mutations, depicted in Figure 2B (sequences in Figure 2—figure supplement 1A). The null mutant has a 4 bp deletion which terminates EphA4 protein within the ligand-binding domain. The truncation mutant terminates the protein at residue 651 (*epha4^Δ651^*), deleting most of the tyrosine kinase domain and all C-terminal domains, and thus completely lacks forward signaling but potentially still activates reverse signaling. The kinase-dead mutant (*ephA4^KD^*) replaces a lysine residue essential for kinase function with methionine. The *epha4^ΔPDZBD^* mutant is truncated at residue 994 which removes the five C-terminal amino acids containing the PDZ binding domain (PDZBD) motif.

**Figure 2—figure supplement 1.**
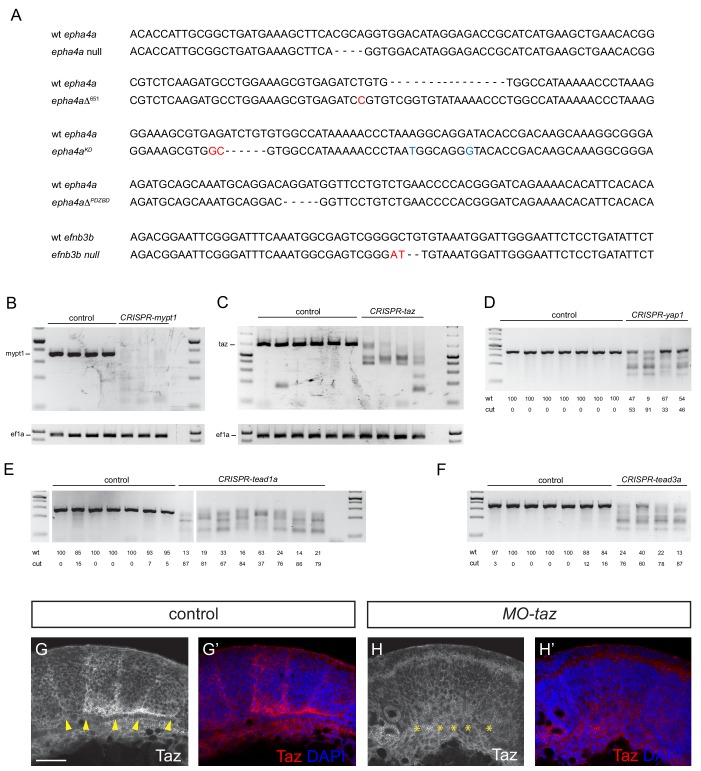
Validation of knockout and knockdown reagents and mutants. (**A**) DNA sequence of the *epha4a* and *efnb3b* mutants. Nucleotides in red indicate small insertions or substitutions introduced at target sites; nucleotides in blue indicate residues that have been introduced by insertion of a template DNA, including the A to T transversion causing the kinase loss-of-function. (**B, C**) PCR of region targeted by multiple gRNAs against *mypt1* (**B**) and *taz* (**C**) suggest that large deletions occur in injected embryos. (**D–F**) T7 endonuclease I digestion of PCR products of regions targeted in *yap1* (**D**), *tead1a* (**E**) and *tead3a* (**F**), with quantitation of undigested (wt) and digested products. Cut bands in DNA from some uninjected embryos in E and F may reflect the presence of SNPs. Five embryos were pooled for each PCR or digestion. (**G, H**) Taz staining in boundaries is lost after *taz* knockdown. Lateral views. Arrowheads indicate Taz^+^ boundary position. Asterisks indicate Taz^-^ boundary position. Scale bar: 50 μm.

**Figure 2—figure supplement 2.**
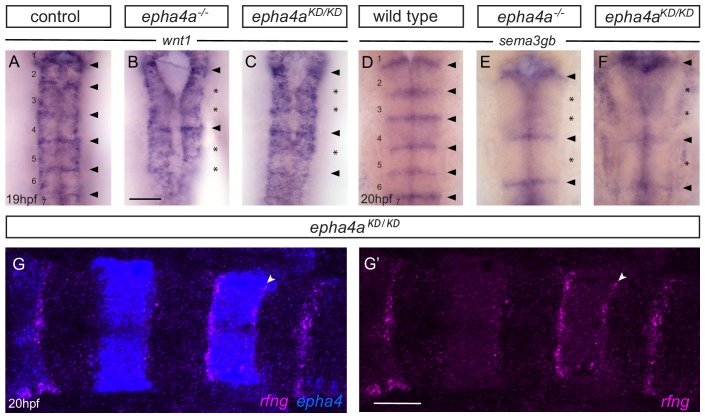
Boundary marker expression in *epha4* mutants. (**A–C**) Expression of *wnt1* is reduced at specific hindbrain boundaries in *epha4^-/-^* (B; 13/13) and *epha4^KD^* (C; 13/15) mutants compared to controls (A; 17/18). Dorsal expression of *wnt1* is not changed in the mutants. (**D–F**) *sema3gb* expression is reduced at specific boundaries in *epha4^-/-^* (E; 14/14) and *epha4^KD^* (F; 17/17) mutants compared to controls (D; 17/17). Arrowheads indicate normal boundary expression of *wnt1* or *sema3gb*, while stars indicate reduction or absence of boundary marker expression. Dorsal views, anterior to the top. (**G, G’**) HCR staining for *rfng* and *epha4a* in *epha4^KD^* mutants reveals that remaining *rfng* expression occurs in *epha4*-expressing cells at the r5/r6 boundary (white arrowhead; 20/20). Dorsal view, anterior to the left. Scale bar: 50 μm.

We found that the null mutant of *epha4* has the same phenotype described previously for *epha4* knockdown, with loss of *rfng* expression at the r2/r3, r3/r4 and r5/r6 borders (Figure 2E; compare with wild type, Figure 2C). Furthermore, expression of other boundary markers, including *wnt1* and *sema3gb*, was disrupted at these borders (Figure 2—figure supplement 2A–F). A milder disruption of *rfng* expression at the r2/r3, r3/r4 and r5/r6 borders is found in *ephrinB3* null mutant embryos (Figure 2D), likely reflecting some functional overlap with *ephrinB2* which is also a ligand for EphA4 (Cooke et al., 2005). The *epha4^Δ651^* truncation mutant was found to have the same loss of *rfng* expression as *epha4* null mutants (Figure 2F), supporting the idea that boundary cell formation is dependent upon EphA4 forward signaling. However, since loss of the cytoplasmic domain of EphA4 might alter its activity as a ligand, this finding does not rule out a contribution of reverse signaling. To address whether kinase-dependent forward signaling is required, we analysed the *epha4^KD^* mutant and found a major decrease, but not complete loss of *rfng* expression at the r2/r3, r3/r4 and r5/r6 borders (Figure 2G). The residual *rfng* expression at the r5/r6 border occurs in *epha4*-expressing cells (Figure 2—figure supplement 2G,G’), arguing against the possibility that it is due to reverse signaling activated by *epha4^KD^*. The presence of some *rfng* expression at segment borders in the *epha4^KD^* mutant suggests that kinase-independent signaling contributes to boundary cell formation. To test whether there is a parallel pathway involving signaling through PDZ domain proteins, we analysed the *epha4^ΔPDZBD^* mutant. We found that there is a mild decrease in *rfng* expression at the r2/r3 and r5/r6 borders, though not at the r3/r4 border (Figure 2H). These findings are consistent with a contribution of PDZBD-dependent signaling to boundary cell formation.

Analysis of *egr2* expression in the e*pha4* null mutant revealed a decrease in sharpness of the r2/r3, r3/r4 and r5/r6 borders, with some *egr2*-expressing cells in the adjacent segments (Figure 2K; compare with control, Figure 2I). The sharpness of the same borders was disrupted in the *ephrinb3*, *epha4^Δ651^* and *epha4^KD^* mutants, though with fewer ectopic *egr2*-expressing cells compared with the null mutant (Figure 2J,L,M). In the *epha4^ΔPDZBD^* mutant there was a decrease in sharpness at the r5/r6 border but only in 30% of the embryos at r2/r3 (Figure 2N). Taken together, these findings suggest that both kinase-dependent and PDZ domain-dependent pathways contribute to upregulation of boundary marker expression, with a stronger input of kinase signaling. There is a correlation between decreased border sharpness and decreased boundary marker expression, suggestive of a mechanistic link.

### Boundary marker expression is regulated by myosin phosphorylation

**Figure 3.**
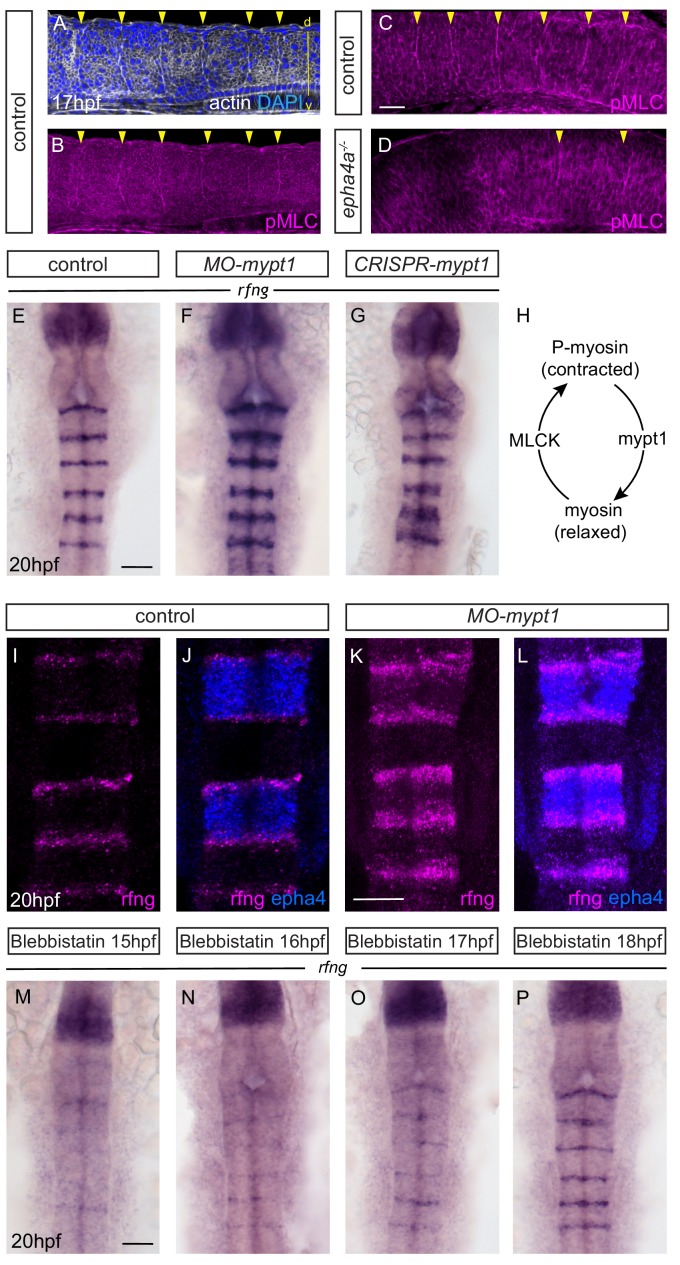
Actomyosin tension regulates boundary expression of *rfng.* (**A, B**) Immunostainings to detect actin (**A**) and pMLC (**B**) which co-localize at segment boundaries. (**C, D**) pMLC is detected at all boundaries in control embryos (C, 37/38) but not at r2/r3, r3/4 and r5/r6 boundaries in *epha4^-/-^* embryos (D, 24/24). Lateral views, anterior to the left. (**E–G**) *rfng* expression is increased in *mypt1* knockdowns (F; 38/45) and embryos injected with CRISPR/Cas9 against *mypt1* (G; 19/26), compared to controls (E; 37/38). (**H**) Depiction of Mypt1 regulating actomyosin tension by dephosphorylating pMLC. (**I–L**) HCR stainings reveal that *rfng* expression in *epha4*-expressing cells in control embryos (I, J; 24/24) is increased after knockdown of *mypt1* (K, L; 28/36). (**M–P**) Myosin II inhibitor blebbistatin suppresses *rfng* transcription when treatment is initiated at 15 hpf (M; 21/21), 16 hpf (N; 25/25) or 17 hpf (O; 23/23), but it is less affected when initiated at 18 hpf (P; reduced in 8/22). (**E–P**) Dorsal views, anterior to the top. Scale bar: 50 μm.

These findings raise the question of how EphA4 forward signaling leads to *rfng* expression at boundaries. EphA4 signaling regulates formation of an actin cable at boundaries, which is first detected at 15 hpf and has been implicated in maintenance of a straight border through actomyosin-dependent generation of cortical tension (Calzolari et al., 2014). Hindbrain boundary cells have a distinct shape from non-boundary cells, which is altered by knockdown of the myosin phosphatase regulator, *mypt1*, that leads to increased phosphorylation of myosin light chain (pMLC) and actomyosin contraction (Figure 3H) (Gutzman and Sive, 2010). Consistent with these findings, we found a higher level of pMLC co-localising with the actin cable at hindbrain borders (Figure 3A,B). Furthermore, pMLC was no longer detected at the r2/r3, r3/r4 and r5/r6 borders in *epha4* null mutants (Figure 3C,D). Surprisingly, we found that knockdown or transient CRISPR/Cas9-mediated knockout of *mypt1* leads to an increase in the level and width of *rfng* expression at hindbrain boundaries (Figure 3E–G). Likewise, *mypt1* knockdown leads to increased expression of *wnt1* and *sema3gb* at boundaries (Figure 3—figure supplement 1A–D). We wondered whether the broader expression of *rfng* after *mypt1* knockdown occurs in regions of forward and/or reverse signaling. By carrying out in situ HCR we found that *rfng* expression spreads only into the *epha4*-expressing domain where forward signaling is occurring (Figure 3I–L). The increased boundary marker expression after *mypt1* knockdown suggests that actomyosin contraction regulates boundary cell formation. To test this, we treated embryos at different time intervals with blebbistatin, an inhibitor of myosin II ATPase activity. We found that blebbistatin treatment from 15 hpf onwards strongly disrupts the upregulation of *rfng* expression at boundaries, with a progressively milder effect on the expression when the treatment is started at later times, and no change detected when treated from 18 hpf (Figure 3M–P). Furthermore, disruption of actin polymerisation by treating embryos with latrunculinB leads to loss of *rfng* expression at boundaries (Figure 3—figure supplement 1E,F).

**Figure 3—figure supplement 1.**
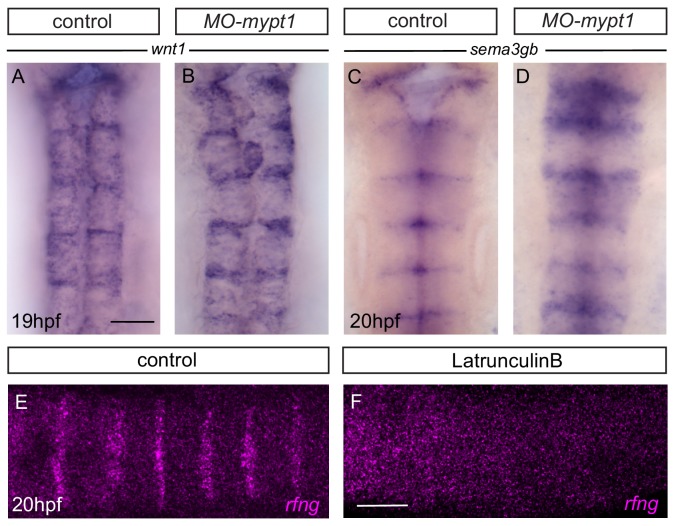
Boundary marker expression after *mypt1* knockdown. (**A, B**) Expression of *wnt1* in control (A; 17/18) increases in *mypt1* knockdown embryos (B; 13/29). (**C, D**) Expression of *sema3gb* in control (C; 17/17) increases in *mypt1* knockdown embryos (D; 12/14). Dorsal views, anterior to the top. (**E, F**) HCR staining reveals that *rfng* in control (**E**) is no longer detected in latrunculinB-treated embryos (F; 12/12). Dorsal views, anterior to the left. Scale bar: 50 μm.

**Figure 4.**
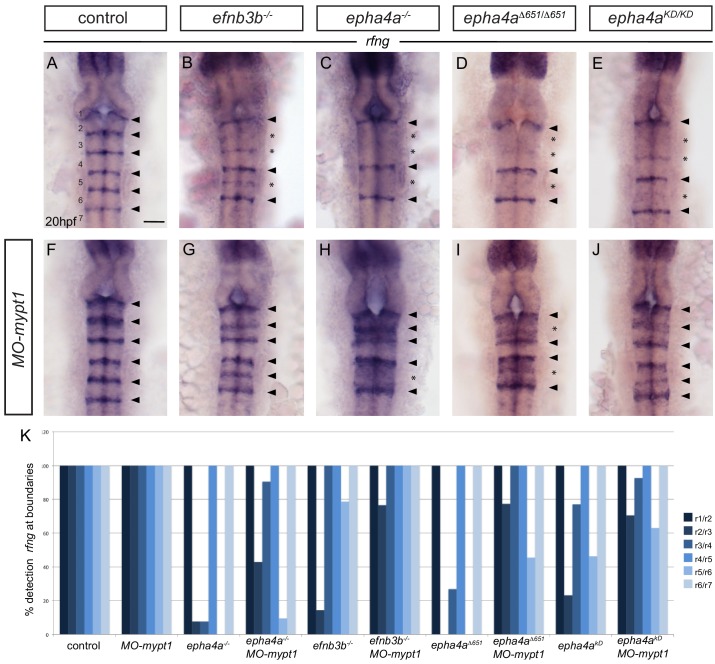
Increased tension selectively restores boundary expression of *rfng* in *epha4* and *ephrinb3* mutants. (**A–K**) Knockdown of *mypt1* increases *rfng* expression at hindbrain boundaries in control embryos (**F**) and restores *rfng* expression at specific boundaries in *ephrinb3^-/-^* (**G**), *epha4^-/-^* (**H**), *epha4^Δ651^* (**I**) and *epha4^KD^* (**J**) mutants compared to uninjected controls (**A–E**). (**K**) Percentage of embryos showing *rfng* expression at the different boundaries for: control (n = 38); *MO-mypt1* (45); *epha4a^-/-^* (13); *epha4a^-/-^MO-mypt1* (42); *efnb3b*^-/-^ (14); *efnb3b*^-/-^ *MO-mypt1* (17); *epha4a^Δ651^* (15); *epha4a^Δ651^ MO-mypt1* (22); *epha4a^KD^* (13); *epha4a^KD^ MO-mypt1* (27). Dorsal views, anterior to the top. Arrowheads indicate normal boundary expression of *rfng*, while asterisks indicate reduction or absence of *rfng* expression at boundaries. Scale bar: 50 μm.

These findings suggest that the induction of hindbrain boundary markers involves increased actomyosin contraction downstream of EphA4 forward signaling. We therefore wondered whether *mypt1* knockdown can rescue the decrease in *rfng* expression that occurs in *epha4* mutants. We found that *mypt1* knockdown in *epha4* null mutants rescues *rfng* expression at the r2/r3 and r3/4 borders, but not at the r5/r6 border (Figure 4C,H; wild type embryos in Figure 4A,F; quantitated in Figure 4K). This suggests that *mypt1* knockdown is increasing residual MLC phosphorylation at the r2/r3 and r3/4 borders in the *ephA4* null mutant, potentially due to other segmentally-expressed Eph receptors, whereas such compensation does not occur at the r5/r6 border. Intriguingly, *mypt1* knockdown rescues *rfng* expression at the r5/r6 border as well as the r2/r3 and r3/r4 borders in the *epha4^Δ651^* and *epha4^KD^* mutants (Figure 4D,E,I–K). This finding suggests that there is some compensation with mutants that have the EphA4 extracellular domain that does not occur when EphA4 protein is completely absent. *mypt1* knockdown rescues *rfng* expression at all hindbrain boundaries in *ephrinB3* null mutants (Figure 4B,G), consistent with residual EphA4 activation by other ephrins.

### taz and tead1a are required for boundary marker expression

**Figure 5.**
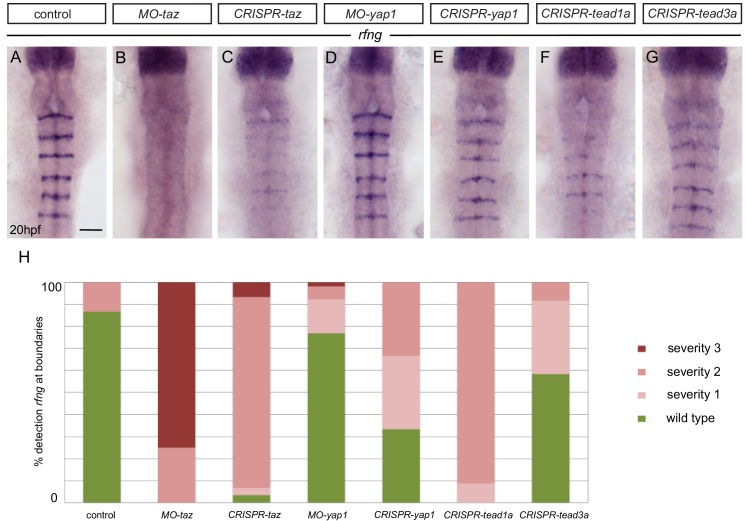
Taz and Tead1a are required for boundary expression of *rfng*. (**A–H**) Hindbrain boundary expression of *rfng* is reduced in *taz* knockdowns (**B**), and in *taz* (**C**) and *tead1a* (**F**) transient knockouts compared to controls (**A**), while *yap1* knockdown (**D**) and *yap1* (**E**) and *tead3a* (**G**) transient knockouts have normal *rfng* expression. (**H**) Scoring of boundary expression of *rfng* in different conditions according to severity levels: wild type = normal expression of *rfng* in all boundaries; severity 1 = general reduction of *rfng* expression levels; severity 2 = partial absence of *rfng* expression leading to discontinuous boundaries; severity 3 = total absence of *rfng* boundary expression. Number of embryos: control (15); MO-*taz* (20); CRISPR-*taz* (30); MO-*yap1* (52); CRISPR-*yap1* (21); CRISPR-*tead1a* (23); CRISPR-*tead3a* (12). Dorsal views, anterior to the top. Scale bar: 50 μm.

Taken together, these findings suggest a model in which EphA4 forward signaling leads to actomyosin contraction that induces boundary marker expression. This raises the question of what pathway links mechanical tension to gene regulation at hindbrain boundaries. To address this, we carried out morpholino-mediated knockdowns of genes that have been implicated in mechanotransduction in other contexts. This screen revealed that knockdown of the *taz* gene disrupts boundary marker expression, including *rfng*, *wnt1* and *sema3gb* (Figure 5A,B,H; Figure 5—figure supplement 1A–D). To test the specificity of the gene knockdown, we carried out transient CRISPR/Cas9-mediated deletions of *taz* and found that this also leads to decreased *rfng* expression at boundaries (Figure 5C,H). In contrast, knockdown or knockout of the related *yap1* gene has little effect on boundary marker expression (Figure 5D,E,H), albeit this could reflect that loss of function is not complete. The strong effect of *taz* loss of function suggests that it has a non-redundant role in upregulation of boundary marker expression.

**Figure 5—figure supplement 1.**
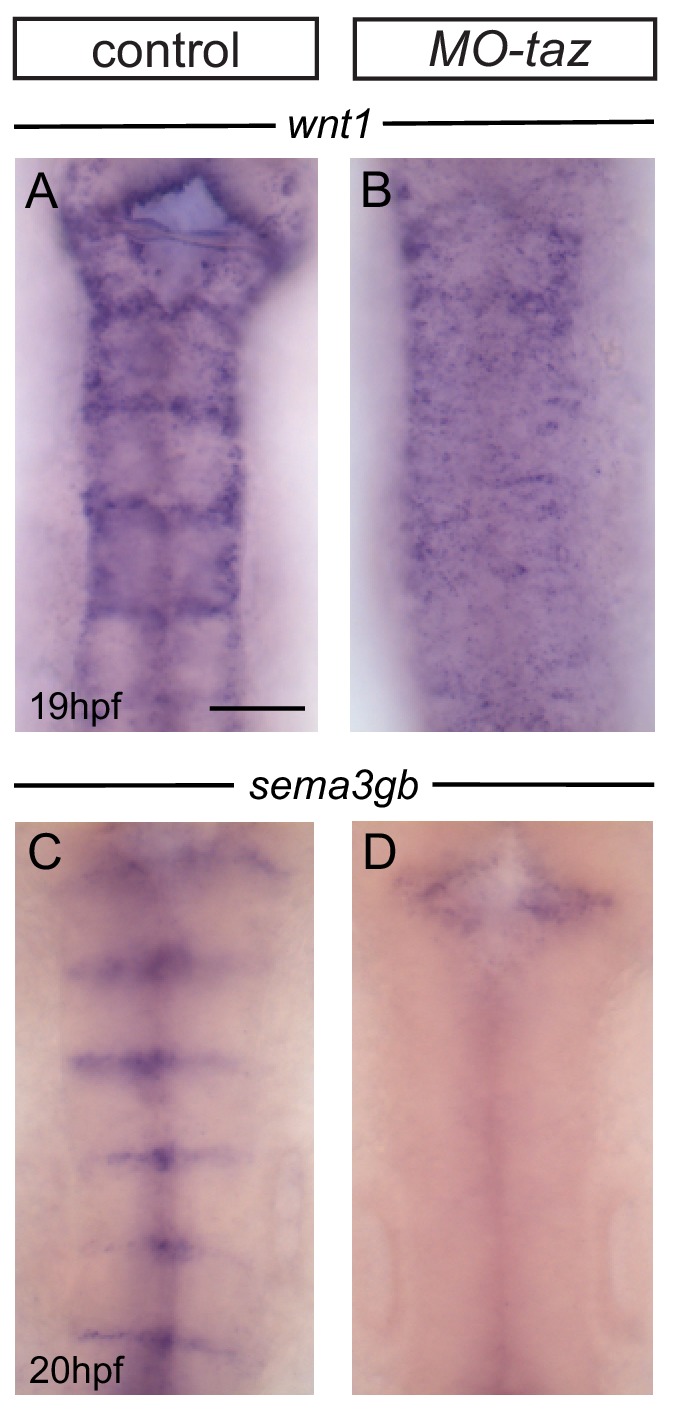
Boundary marker expression after *taz* knockdown. (**A, B**) Boundary expression of *wnt1* in control (A; 17/18) is no longer detected in *taz* knockdown embryos (B; 12/12). (**C, D**) Boundary expression of *sema3gb* in control (C; 17/17) is no longer detected in *taz* knockdown embryos (D; 19/19). Boundary expression of both markers is lost following *taz* knockdown. Dorsal views, anterior to the top. Scale bar: 50 μm.

Taz and Yap have been intensively studied as components of a pathway which links mechanical tension to the regulation of cell proliferation (Elbediwy et al., 2016; Gaspar and Tapon, 2014; Halder et al., 2012; Low et al., 2014). In addition, Taz and Yap have been implicated in the maintenance of stem cells or regulation of cell differentiation in specific tissues (Chen et al., 2019; Isomursu et al., 2019; Luxenburg and Zaidel-Bar, 2019; Mo et al., 2014; Panciera et al., 2017; Varelas, 2014). Mechanical cues or other inputs lead to the translocation of Yap/Taz protein from cytoplasm to the nucleus, where they can interact with Tead family transcription factors to regulate specific gene expression. Gene expression studies have found that two tead family members, *tead1a* and *tead3*, are widely expressed in the nervous system, with segmental regulation of the level of expression (Thisse et al., 2001). To determine whether Taz acts together with these Tead family transcription factors to regulate boundary gene expression, we carried out transient Crispr-mediated knockouts. We found that knockout of t*ead1a*, but not of *tead3*, leads to a decrease in *rfng* expression (Figure 5F–H). Since the decrease in *rfng* expression was less than occurs following *taz* knockout, there may be partial redundancy with other *tead* family members.

### Myosin regulation downstream of EphA4 regulates Taz localisation

**Figure 6.**
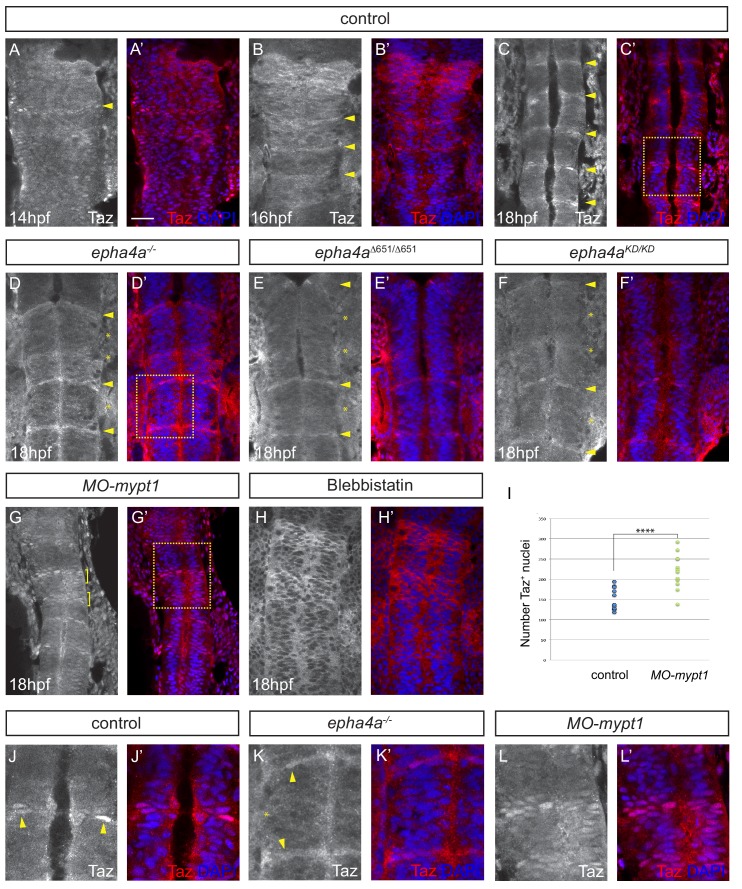
Eph-ephrin signalling and actomyosin tension regulate Taz nuclear localization. (**A–C**) Time course of the localization of Taz protein. Nuclear localization of Taz starts to be detected in hindbrain boundaries at 14 hpf (**A, A’**). Several boundaries have elevated nuclear Taz at 16 hpf (**B, B’**), and nuclear Taz is present in all boundaries at 18 hpf (**C, C’**). (**D–F**) Nuclear Taz is reduced at r2/r3, r3/r4 and r5/r6 boundaries in *epha4* null (**D, D’**), epha4*^Δ651^* (**E, E’**) and *epha4a^KD^* mutants (**F, F’**) at 18 hpf. (**G, G’**) Ectopic cells with elevated nuclear Taz are observed at 18 hpf after *mypt1* knockdown. (**H, H’**) Blebbistatin treatment inhibits the nuclear accumulation of Taz at boundaries. (**I**) Quantitation of number of nuclei with Taz staining in controls (n = 12) and *mypt1* knockdowns (n = 13) (****p<0.0001). (**J–L**) Higher magnification images corresponding to boxed areas in C’, D’ and G’. Dorsal views, anterior to the top. Arrowheads indicate boundary position; asterisks indicate boundaries with reduced nuclear Taz; brackets indicate expansion of nuclear Taz staining. Scale bar: 30 μm.

To determine whether EphA4 signaling and actomyosin contraction acts by regulating the subcellular localisation of Taz protein, we first carried out immunostaining studies during normal hindbrain development. We found an increased level of Taz, accompanied by nuclear localisation in some cells, at hindbrain boundaries, starting at 14 hpf and becoming more prominent at 18 hpf (Figure 6A–C,J; Figure 6—figure supplement 1). Since not all cells at boundaries have nuclear Taz, there may be a dynamic regulation of subcellular localisation. These observations are consistent with evidence that the actin cytoskeleton can regulate the stability as well as nuclear localisation of Taz (Dupont et al., 2011). To determine whether Taz localisation is regulated downstream of EphA4, we carried out immunostaining in *epha4* null mutants. We found that there is no longer nuclear Taz staining at the r2/r3, r3/r4 and r5/r6 borders, whereas the increased level of Taz and nuclear localisation remains at the r1/r2, r4/5 and r6/r7 borders, where boundary marker expression occurs in *epha4* mutants (Figure 6D,K; Figure 6—figure supplement 1). To determine whether Taz localisation is regulated by forward signaling, we analysed *epha4^Δ651^* and *epha4^KD^* mutants and found that nuclear Taz was no longer detected at the r2/r3, r3/r4 and r5/r6 borders (Figure 6E,F; Figure 6—figure supplement 1). These findings suggest that forward but not reverse signaling leads to nuclear translocation of Taz. To test whether Taz localisation is influenced by myosin phosphorylation, we carried out *mypt1* knockdown and found that this leads to an increase in the number of cells with nuclear Taz at segment borders (Figure 6G,I,L). This finding is consistent with the observation of an increased number of cells expressing *rfng* following *mypt1* knockdown. Finally, we analysed the effect of decreasing myosin II function by treating embryos with blebbistatin and found a decrease in nuclear localisation of Taz (Figure 6H).

**Figure 6—figure supplement 1.**
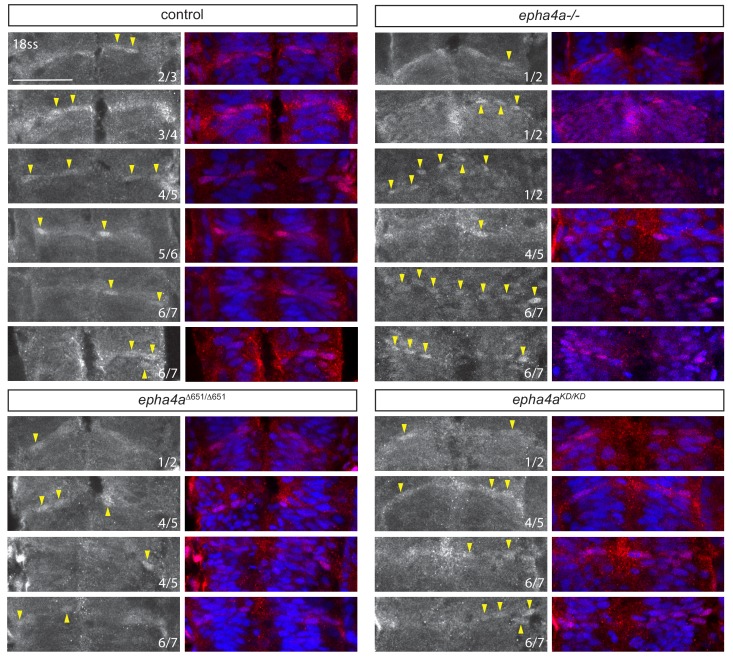
Taz localisation in hindbrain boundaries. Additional examples of Taz nuclear accumulation in boundaries at different conditions. In control embryos, Taz accumulates in the nuclei of some cells in all boundaries, but only in the r1/r2, r4/r5 and r6/r7 boundaries in *epha4a* null, *epha4a*^Δ651^ and *epha4a^KD^* mutants. The numbers indicate which boundary is shown and arrowheads indicate Taz+ nuclei. Scale bar: 50 μm.

**Figure 6—figure supplement 2.**
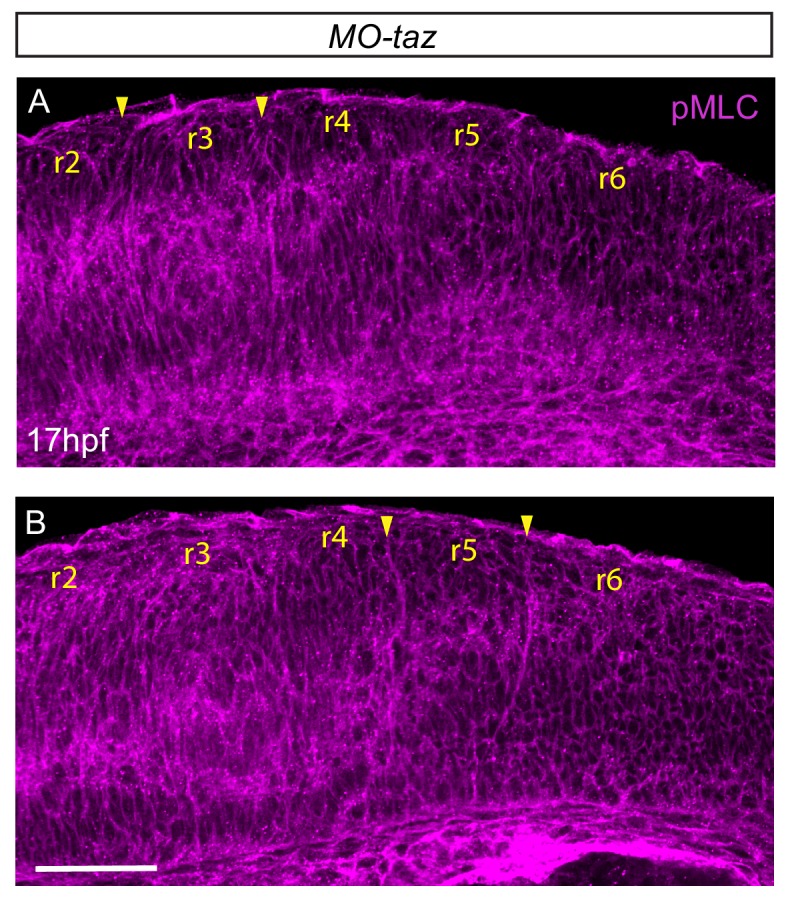
pMLC after *taz* knockdown. (**A, B**) *taz* knockdown embryos immunostained to detect pMLC, shown in two different confocal planes. Increased pMLC is detected at hindbrain boundaries, suggesting that myosin phosphorylation does not require Taz function. Lateral views, anterior to the top. Scale bar: 50 μm.

The *Drosophila* homologue of Yap/Taz, Yorkie, can increase myosin activity and tension independently of its function as a transcription co-factor (Xu et al., 2018). We therefore wondered whether Taz is required for actomyosin regulation in the hindbrain. To address this question, we analysed MLC phosphorylation following knockout of Taz, and found that pMLC is still elevated at hindbrain boundaries (Figure 6—figure supplement 2). Taken together, these findings support a model in which EphA4 activation leads to actomyosin phosphorylation and contraction at segment borders, which in turn increases nuclear localisation of Taz and boundary marker expression.

## Discussion

**Figure 7.**
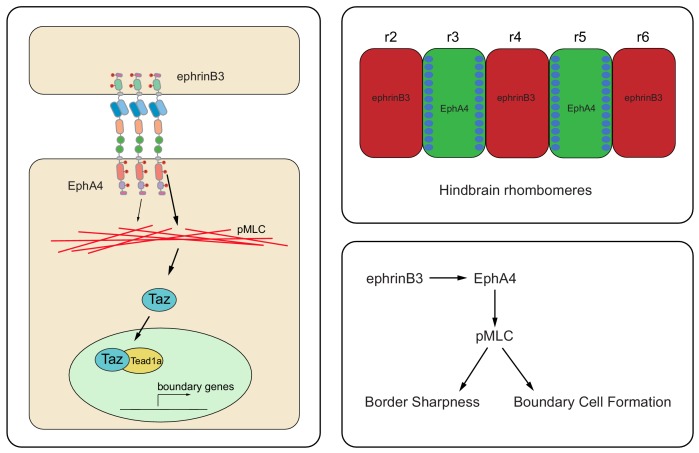
Model of EphA4 signaling and hindbrain boundary cell induction. The diagram on the left depicts the activation of EphA4 forward signaling by ephrinB3, which through kinase-dependent and PDZ binding domain-dependent pathways increases the level of pMLC. pMLC promotes actomyosin contraction and translocation of Taz to the nucleus, where it interacts with Tead1a to upregulate boundary-specific gene expression. As a consequence, boundary cells form in EphA4-expressing cells at the borders of r3 and r5 (top right). By acting through pMLC, EphA4 signaling couples boundary cell formation to the maintenance of border sharpness (bottom right).

A key concept that came from early studies of compartment boundaries is that sharp borders enable the correct organisation of signaling centres (Dahmann and Basler, 1999). However, it remains unclear whether or how border sharpening and boundary cell formation are coordinated. We have studied this in the vertebrate hindbrain, in which segment borders are sharpened and boundary cells form that act as a signaling centre. We show that forward signaling of EphA4, which regulates myosin light chain phosphorylation that increases cortical tension, is required both for border sharpening and for hindbrain boundary cell formation. Furthermore, increasing myosin II phosphorylation by knockdown of *mypt1* increases boundary marker expression, whereas inhibition of myosin II function or actin polymerization blocks boundary marker expression. We show that EphA4 forward signaling and myosin phosphorylation induce nuclear translocation of Taz, which together with Tead1a regulates boundary marker expression (Figure 7). It is likely that activation of other Eph receptors underlies increased pMLC and boundary gene expression at the r1/r2, r5/r6 and r6/r7 borders that are not disrupted in the EphA4 mutants; for example, EphB4 which underlies cell segregation and has complementary expression to ephrinb2a (Chan et al., 2001; Cooke et al., 2001; Cooke et al., 2005). Interestingly, the *rac3b* gene which is adjacent to *rfng* is co-regulated in hindbrain boundary cells, and knockdown of *rac3b* disrupts actomyosin cable formation and border sharpness (Letelier et al., 2018). Taken together with our findings, this suggests that Eph signaling initiates boundary gene expression by increasing actomyosin contraction, which is then maintained in a positive feedback loop through expression of *rac3b*. Since increased tension underlies the maintenance of a straight border (Calzolari et al., 2014), cell segregation and boundary cell formation are coupled, thus ensuring that boundary cells are organised at a sharp border (Figure 7).

### EphA4 signaling and boundary cell formation

By generating a series of point and deletion mutants of *epha4*, we find that forward signaling is essential for boundary marker expression, with a strong input of kinase-dependent signaling and lesser input of PDZ binding domain dependent signaling. These findings are consistent with studies of the regulation of cell repulsion and cortical tension by Eph receptor signaling (Canty et al., 2017; Fagotto et al., 2013; O'Neill et al., 2016; Rohani et al., 2011; Rohani et al., 2014; Taylor et al., 2017). Cell repulsion and tension are regulated by increased Rho activity, which leads to myosin light chain phosphorylation and actomyosin contraction at borders where Eph receptor activation is occurring (Fagotto et al., 2013; Rohani et al., 2014). Multiple kinase-dependent pathways have been found to link Eph receptor forward signaling to Rho activation (Jørgensen et al., 2009; Kania and Klein, 2016; Pasquale, 2008). Eph kinase-independent signaling can also lead to cell repulsion and segregation (Taylor et al., 2017) and can activate Rho, for example through binding of Dishevelled to the PDZ domain binding motif of Eph receptors (Tanaka et al., 2003). Such kinase-independent signaling leads to a less sustained cell repulsion response than occurs when Eph kinase function is intact (Taylor et al., 2017). Taken together, these findings reveal some functional overlap between kinase- and PDZBD-dependent signaling, with a greater role of Eph kinase-activated pathways both in cell segregation and boundary cell induction.

Previous studies had not resolved whether boundary cells form on one or both sides of the interface of hindbrain segments. We find that for r3 and r5 they form on one side of each interface, in the *epha4*-expressing cells, and this is because they are induced by forward and not by reverse signaling. This finding is consistent with evidence that although reverse signaling can trigger cell repulsion, forward signaling leads to much stronger cell repulsion and actomyosin contraction and thus has a dominant role in cell segregation and border sharpening (Canty et al., 2017; Fagotto et al., 2013; O'Neill et al., 2016; Rohani et al., 2011; Rohani et al., 2014; Taylor et al., 2017; Wu et al., 2019). However, *rfng* expression is also detected in some cells adjacent to r3 or r5 that are not expressing *epha4*, in particular at the r5/r6 border. The finding that such expression adjacent to r3 and r5 does not occur in *epha4^Δ651^* or *epha4^KD^* mutants argues against the possibility that any MLC phosphorylation induced by reverse signaling upregulates boundary marker expression. An alternative explanation is that *rfng*-expressing cells in r6 derive from intermingling of boundary cells across the segment border. This explanation requires that *epha4* expression is downregulated in r5 cells that intermingle into adjacent segments, and indeed recent work has found dynamic regulation of r3 and r5 cell identity following intermingling (Addison et al., 2018).

The decrease in boundary marker expression in *epha4* mutants is partially rescued by *mypt1* knockdown, suggesting that there is residual activation of myosin II at specific borders, perhaps due to other Eph-ephrin pairs. Intriguingly, the r5/r6 boundary was not rescued in *epha4* null mutants, but was in *epha4^KD^* and *epha4^Δ651^* mutants. Since the *epha4^Δ651^* mutant lacks forward but not reverse signaling, whereas the *epha4* null mutant lacks both, this could suggest that reverse signaling into r6 cells can induce boundary marker expression when tension is amplified by *mypt1* knockdown. Indeed, although forward signaling underlies actomyosin contraction in border sharpening, it remains possible that reverse signaling also leads to MLC phosphorylation, which can lead to boundary marker expression when increased by *mypt1* knockdown. However, *rfng* expression spreads into r5 but not r6 after *mypt1* knockdown, arguing against this idea. As some EphA and EphB receptors can form heteromers (Fox and Kandpal, 2011), an alternative explanation is that truncated or kinase-dead EphA4 enables activation of another Eph receptor by ephrinB3. Indeed, EphB4, which has a low affinity for ephrinB3 (Noberini et al., 2012), is expressed in r5 and r6 and regulates cell segregation (Cooke et al., 2001).

Intriguingly, *mypt1* knockdown leads to spreading of *rfng* expression into r3 and r5, several cell diameters away from the segment border, whereas EphA4 is assumed to be activated at the border. One potential explanation is suggested by the finding that during liver development ephrin-expressing cells extend processes into Eph-expressing territory and thus regulate cell behaviour away from the border (Cayuso et al., 2016). If this also occurs in the hindbrain, it could lead to weak EphA4 activation a few cell diameters from the border, which can upregulate *rfng* expression when tension is amplified by *mypt1* knockdown. Another possible mechanism is through secretion of ephrin-containing exosomes, which have been detected in cell culture (Gong et al., 2016), though it is not known whether exosomes mediate ephrin signaling in vivo.

### Regulation of cell identity by Taz activity

There is increasing evidence for roles of Yap/Taz activity in maintaining stem cells, or in some tissues in promoting their differentiation to specific derivatives (Kumar et al., 2017; Mo et al., 2014; Panciera et al., 2017; Varelas, 2014). In some contexts, nuclear translocation of Yap/Taz protein is regulated by forces originating from interaction of cells with extracellular matrix, from stretching, shearing and compression of cells, and from actomyosin contractility within the cell (Elbediwy et al., 2016; Halder et al., 2012; Low et al., 2014; Sun and Irvine, 2016; Varelas, 2014). Hindbrain boundary cells are neural progenitors that are prevented from differentiating through Notch activation, which is promoted by Rfng (Cheng et al., 2004), thus maintaining the boundary signaling centre (Terriente et al., 2012). Activation of Taz by actomyosin contraction therefore leads to the formation and maintenance of these specialised progenitors in part through regulation of Notch pathway activity. Likewise, an interplay between Yap/Taz and the Notch pathway that maintains progenitors has been found in other tissues (reviewed by Totaro et al., 2018). For example, Yap/Taz maintains epidermal stem cells by inhibiting Notch signaling through regulation of Notch pathway components (Totaro et al., 2017). In another example, the contractility of muscle cells activates Yap, which upregulates Jag2 expression, leading to Notch activation in neighbours that inhibits their differentiation (Esteves de Lima et al., 2016).

Yap and Taz also have important roles in growth control in which genes that drive proliferation are upregulated by nuclear localisation of Yap/Taz, which is inhibited by activation of the Hippo pathway (Gaspar and Tapon, 2014; Halder and Johnson, 2011; Low et al., 2014). Since cortical tension leads to nuclear localisation of Taz at hindbrain boundaries, this raises the question of whether actomyosin contraction increases cell proliferation in addition to inducing boundary marker expression. Studies in chick argue against this idea as hindbrain boundaries have a lower proliferation rate than segment centres (Guthrie et al., 1991; Peretz et al., 2016), reflecting their role as a pool of neurogenic stem cells. However, recent work has found two-fold greater proliferation at boundaries than segment centres at late stages in the zebrafish hindbrain (after 26 hpf), which depends upon Yap/Taz and Tead activity (Voltes et al., 2019). Importantly, this work found that the Yap/Taz-Tead pathway is activated by actomyosin tension at boundaries and therefore increases cell proliferation downstream of Eph-ephrin signaling. Yap/Taz activation and cell proliferation declines by 40 hpf, concommitant with a switch of boundary cells to neurogenesis (Voltes et al., 2019). However, since this study used reporters that detected Tead activity only after 20 hpf, it did not test an earlier role in boundary cell specification, which we find occurs prior to 18 hpf. Taken together, these findings suggest stage-specific functions of Yap/Taz activity in cell specification and proliferation at hindbrain boundaries.

### Concluding perspectives

The mechanical regulation of gene expression enables an interplay between morphogenesis and cell identity that contributes to tissue patterning (Chan et al., 2017; Kim et al., 2018; Xia et al., 2019). The transcriptional control of cell differentiation leads to differential expression of mediators of morphogenesis, creating mechanical forces which can in turn feed back on the specification of cell identity. In the hindbrain, *epha4* expression is regulated by *krox20* (Theil et al., 1998), such that cell segregation and border sharpening is coupled to segmental identity (Tumpel et al., 2009). Mechanical forces regulated by EphA4 signaling in turn lead to the specification of boundary cell fate, thus ensuring correct organisation of signaling centres. There is increasing evidence for roles of Eph receptors and ephrins in the regulation of cell differentiation through a diversity of pathways (Laussu et al., 2014; Wilkinson, 2014). In some cases, Eph receptor activation seems to be deployed to only regulate cell differentiation, by acting through pathways distinct from those that underlie cell segregation. For example, Eph activation regulates cell fate choices in Ciona by antagonising Fgf signaling through inhibition of the MAPK pathway (Picco et al., 2007; Stolfi et al., 2011). It will be important to understand how Eph signaling has these distinct functions in cell segregation and regulation of cell differentiation in different contexts. It will also be interesting to determine whether it has broader roles in activating the Yap/Taz pathway to couple border formation and the control of cell identity. In particular, it may be fruitful to explore this in vertebrate development, in which Eph signaling underlies the formation and maintenance of sharp borders in many tissues (Batlle and Wilkinson, 2012; Cayuso et al., 2015; Fagotto et al., 2014). Since the principal mechanisms that drive border sharpening are contact repulsion and cortical tension that require actomyosin contraction (Canty et al., 2017; Fagotto et al., 2013; O'Neill et al., 2016; Rohani et al., 2011; Rohani et al., 2014; Taylor et al., 2017), this raises the question as to whether the pathway uncovered here in the hindbrain is deployed in other tissues to regulate gene expression through Yap/Taz activation.

## Materials and methods

**Key resources table:** 

Reagent type (species) or resource	Designation	Source or reference	Identifiers	Additional information
Recombinant DNA reagent	rfng (zebrafish, *Danio rerio*) in situ hybridisation probe	Cheng et al., 2004, PMID: 15068793		
Recombinant DNA reagent	wnt1 (zebrafish) in situ hybridisation probe	Molven et al., 1991, PMID: 2009859		
Recombinant DNA reagent	sema3gb (zebrafish) in situ hybridisation probe	Terriente et al., 2012, PMID: 22764046		
Recombinant DNA reagent	egr2b (zebrafish) in situ hybridisation probe	Oxtoby and Jowett, 1993, PMID: 8464695		
Sequence-based reagent	rfng (zebrafish) HCR probe	Molecular Instruments		Designed and supplied by Molecular Instruments
Sequence-based reagent	wnt1 (zebrafish) HCR probe	Molecular Instruments		Designed and supplied by Molecular Instruments
Sequence-based reagent	epha4 (zebrafish) HCR probe	Molecular Instruments		Designed and supplied by Molecular Instruments
Antibody	anti-pMLC (rabbit polyclonal)	Cell Signalling Technology	Cat # 3671 RRID:AB_330248	1:200
Antibody	anti-Taz (rabbit monoclonal)	Cell Signalling Technology	Cat # D24E4 RRID:AB_10950494	1:200
Antibody	anti-EphA4 (rabbit polyclonal)	Irving et al., 1996, PMID: 8575627		1:450
Chemical compound, drug	phalloidin-atto 647N	Sigma-Aldrich	Cat # 65906	1:250
Chemical compound, drug	blebbistatin	Sigma-Aldrich	Cat # B0560	12.5 μM
Chemical compound, drug	latrunculinB	Sigma-Aldrich	Cat # 428020	50 nM
Sequence-based reagent	morpholino to block mypt1	Gutzman and Sive, 2010, PMID: 20147380		ATTTTTTGTGACTTACTCAGCGATG
Sequenced-based reagent	morpholino to block taz	Hong et al., 2005, PMID: 16099986		CTGGAGAGGATTACCGCTCATGGTC
Gequenced-based reagent	morpholino to block yap1	Skouloudaki et al., 2009, PMID: 19439659		AGCAACATTAACAACTCACTTTAGG
Genetic reagent (*D. rerio*)	epha4a null mutant	This study		Sequence in Figure 2—figure supplement 1
Genetic reagent (*D. rerio*)	epha4a Δ651 mutant	This study		Sequence in Figure 2—figure supplement 1
Genetic reagent (*D. rerio*)	epha4a KD mutant	This study		Sequence in Figure 2—figure supplement 1
Genetic reagent (*D. rerio*)	epha4a ΔPDZBD mutant	This study		Sequence in Figure 2—figure supplement 1
Sequence-based reagent	genomic target of gRNA to create epha4a null mutant	This study		GGCTGATGAAAGCTTCACGC
Sequence-based reagent	forward primer to screen for epha4a null mutant	This study		GCTCCGCAGTACATTTTAGGG
Sequence-based reagent	reverse primer to screen for epha4a null mutant	This study		GTCTTTCCTCTCACAGTGGGA
Sequence-based reagent	genomic target of gRNA to create epha4a Δ651 mutant	This study		GGAAAGCGTGAGATCTGTG
Sequence-based reagent	forward primer to screen for epha4a Δ651 mutant	This study		CCCTTCACATACGAGGACCCC
Sequence-based reagent	reverse primer to screen for epha4a Δ651 mutant	This study		GCTCGCTCACATTCAACACA
Sequence-based reagent	genomic target of gRNA to create epha4a KD mutant	This study		GGAAAGCGTGAGATCTGTG
Sequence-based reagent	Donor oligonucleotide to create epha4a KD mutant	This study		AAGATGCCTGGAAAGCGTGAaATtTGcGTGGCCATAAAAACCCTAAtGGCAGGgTACACCGACAAGCAAAGGCG
Sequence-based reagent	forward primer to screen for epha4a KD mutant	This study		CCCTTCACATACGAGGACCCC
Sequence-based reagent	reverse primer to screen for epha4a KD mutant	This study		GCTCGCTCACATTCAACACA
Sequence-based reagent	genomic target of gRNA to create epha4a ΔPDZBD mutant	This study		GCAGCAAATGCAGGACAGGA
Sequence-based reagent	forward primer to screen for epha4a ΔPDZBD mutant	This study		AGTTCTCCCCCTCAAACAAAA
Sequence-based reagent	reverse primer to screen for epha4a ΔPDZBD mutant	This study		CAGTACAGCGCTAAACGATCC
Sequence-based reagent	genomic targets of gRNAs to create deletions in mypt1	This study		GGTACGGTACGAAAGAGAGG ACGAAGGTGAAGTTCGACGA GGAACGAGCAGTTAAAGCGC CTGCTCGAGCGGAGACACGG TGGCGGACGCCAAGCAGAAG
Sequence-based reagent	forward primer to screen for deletions in mypt1	This study		CGACGTAACCAGGTTTGTTCA
Sequence-based reagent	reverse primer to screen for deletions in mypt1	This study		ACATTGGCGTAGTTGATGTCG
Sequence-based reagent	genomic targets of gRNAs to create deletions in yap1	This study		CTCAACCTCATCGGCACGGA CCCGAACATGGACGATCTGG AAGAGCCTCCAGATCGGTCT
Sequence-based reagent	forward primer to screen for deletions in yap1	This study		GCCGGACACAGAACATCTTTT
Sequence-based reagent	reverse primer to screen for deletions in yap1	This study		CTGTTTGTGGTTTCTGAGGGG
Sequence-based reagent	genomic targets of gRNAs to create deletions in taz	This study		CAAAGACCTGGACACGGATC GAGATGGCCTTCACCCCCAA GGAGACTCCACTCCCACACC
Sequence-based reagent	forward primer to screen for deletions in taz	This study		AAAACTCTCCAAACTCCACGC
Sequence-based reagent	reverse primer to screen for deletions in taz	This study		CCGTGTTCAATACTCATTCCCC
Sequence-based reagent	genomic targets of gRNAs to create deletions in tead1a	This study		TAAGCCCATGGACAATGACG TGACATTGAGCAGAGCTTTC CATTCTCTCAATGTCCTCCC
Sequence-based reagent	forward primer to screen for deletions in tead1a	This study		GTCAGTGTGCCTTGAGTTCTC
Sequence-based reagent	reverse primer to screen for deletions in tead1a	This study		ATTTTGCCCTCATCAGACAGG
Sequence-based reagent	genomic targets of gRNAs to create deletions in tead3	This study		CATTGAACAAAGCTTCCAGG CTGAGAGGATGATCTTTCTG ATGGACAAAACCGGAATGGA
Sequence-based reagent	forward primer to screen for deletions in tead3	This study		GAGCCGCCACCATTGCAG
Sequence-based reagent	reverse primer to screen for deletions in tead3	This study		TAGCTCTGACTAACGTGGGTG
Sequence-based reagent	RVD sequences of ephrinb3b TALENs	This study		Left: NG HD NN NN NN NN NI NG NG NG HD NI NI NI NG NN NN HD Right: HD NI NN NN NI NN NI NI NG NG HD HD HD NI NI NG HD HD NI NG

### Maintenance of zebrafish strains

Zebrafish embryos were raised at 28.5°C as described (Westerfield, 2007). Embryos were staged according to morphological criteria (Kimmel et al., 1995).

### Morpholino knockdown

Antisense morpholino oligonucleotides (Gene Tools) were injected into one-cell-stage embryos. All injections were done in *p53* homozygote mutants or in combination with a *p53* morpholino to inhibit the off-target effects mediated by activation of pro-apoptotic pathways (Gerety and Wilkinson, 2011; Robu et al., 2007). The antisense morpholinos used were a splice-blocking morpholino against *mypt1* (Gutzman and Sive, 2010) and *yap1* (Skouloudaki et al., 2009), and a translation-blocking morpholino against *taz* (Hong et al., 2005); the sequences are in the Key Resources Table. 4 ng of morpholino were injected in all cases except for MO-taz, for which 2.5 ng were injected. *mypt1* MO has the same effects on cell shape and increased pMLC as *mypt1* mutant embryos (Gutzman and Sive, 2010). *yap1* MO has been validated by analysis of splicing and in rescue experiments (Fukui et al., 2014). taz MO leads to decreased Taz protein (Figure 2—figure supplement 1G–H). In addition, the phenotypic effects described in this study for MO-mediated knockdowns have been validated by generation and analysis of Crispr-mediated transient knockouts.

### Pharmacological treatments

Embryos were dechorionated and treated at the specified stages with 12.5 μM blebbistatin or 50 nM LatrunculinB in Danieau’s solution. Embryos were fixed in 4% paraformaldehyde (PFA) and processed for immunostaining or in situ hybridization.

### Generation of mutants

All injections were done in one-cell stage embryos. *ephrinb3b* mutants were generated using TALENs designed and constructed as previously outlined (Cermak et al., 2011). Plasmids used in the construction process (Golden Gate TALEN and TAL Effector Kit 1.0, #1000000016) as well as pCS2TAL3-DD and pCS2TAL3-RR destination vectors (#37275 and #37276) (Dahlem et al., 2012) were obtained from Addgene. TAL effector domains and FokI nuclease were cloned into these destination vectors to form the final pCS2-TAL vector for each TALEN, from which mRNA was synthesised using the SP6 mMessage mMachine kit (Life Technologies). Embryos were injected with equal amounts (100–300 pg) of RNA encoding each of the left and right TALEN arms. A founder with a frame shift (4 bp deletion and 2 bp insertion) that truncates the protein at residue five was used to raise the *ephrinb3b* mutant line. RVD sequences of *ephrinb3b* TALENs are in the Key Resources Table.

Point and truncated mutants of *epha4a* were generated by CRISPR/Cas9. For this, oligonucleotides targeting different *epha4a* sequences were cloned into the pDR274 plasmid for sgRNA production (Hwang et al., 2013; #42250 Addgene). In vitro synthesis of the sgRNA was done using the T7 RiboMAX Large Scale RNA Production System (#P1300 Promega). Embryos were injected with 200–300 pg gRNAs and 1.6 ng EnGen Cas9 protein (#M0646M NEB). The target and gRNA sequences, and mutations generated, are given in the Key Resources Table and Figure 2—figure supplement 1A. Immunostaining for EphA4 confirmed a complete absence of protein in homozygous null embryos.

To introduce the K658M mutation in the kinase domain of EphA4a, sgRNA and Cas9 protein were co-injected with a 74 bp donor oligonucleotide (Key Resources Table) containing three silent mutations at the gRNA target site, the K658M mutation and an additional silent mutation that generated an RsaI restriction site. Mutations were identified by amplicon restriction using restriction enzymes or T7 endonuclease I (#M0302L NEB) and verified by sequencing. A fish was identified carrying the K658M mutation together with a 6 bp deletion affecting three additional residues (649-651) in the kinase domain.

For the transient CRISPR knockouts of *mypt1*, *yap1*, *taz*, *tead1a* and *tead3a*, 3 to 5 crRNAs targeting the same gene (Key resources table) were obtained from Integrated DNA Technologies Inc (IDT, Iowa, USA). crRNAs were annealed with equimolar amounts of tracRNA and 100 to 150 pg of each gRNA were co-injected with Cas9 protein. The generation of deletions was validated by PCR and in some cases by detection of mismatches by T7 endonuclease I digestion (Auer et al., 2014) (Figure 2—figure supplement 1B–F).

### Immunohistochemistry and in situ hybridization

For immunohistochemistry, embryos were fixed in 4% PFA for 2 hr and processed using standard methods. For anti-Taz stainings, antigen retrieval was carried out by heating fixed embryos at 90°C for 20 min in 150 mM Tris-HCl pH 9.5, which were then rinsed and treated with 0.025 U/ml DNAse1 for 75 min at 37°C prior to staining. Samples were imaged using a Leica SP5 confocal microscope. Antibodies against Taz and pMLC were from Cell Signalling Technology (#D24E4 and 3671, respectively). Anti-EphA4 was described previously (Irving et al., 1996). Actin was detected using phalloidin-atto 647N. Nuclear Taz staining was measured using Volocity software (Improvision) and statistical analysis carried out using unpaired two-tailed Student’s t-test.

For in situ hybridization, embryos were fixed in 4% PFA overnight at 4°C and kept in methanol at −20°C prior to processing. The probes used have been previously described: *egr2b* (Oxtoby and Jowett, 1993), *rfng* (Cheng et al., 2004), *wnt1* (Molven et al., 1991), *sema3gb* (Terriente et al., 2012). Digoxigenin-UTP labelled riboprobes were synthesised and in situ hybridization performed as previously described (Xu et al., 1994). After BCIP/NBT color development, embryos were re-fixed, cleared in 70% glycerol/PBS, and mounted for imaging using a Zeiss Axioplan2 with Axiocam HRc camera. In some experiments, *rfng, wnt1* and *epha4a* transcripts were detected by hybridization chain reaction (HCR) using reagents obtained from Molecular Instruments (In Situ HCR v3.0) and the method described by Choi et al. (2016).

## Data Availability

All data generated or analysed during this study are included in the manuscript and supporting files.

## Additional files

Transparent reporting form
