# Supplementary material for: Actomyosin regulation by Eph receptor signaling couples boundary cell formation to border sharpness
Source: eLife. 2019 Sep 10;8:e49696. doi: 10.7554/eLife.49696 (PMC6739871; doi:10.7554/eLife.49696)
Supplement: Peer review history

# Decision letter

**DOI:** 10.7554/eLife.49696.017

**Affiliations:** California Institute of TechnologyUnited States; Friedrich Miescher Institute for Biomedical ResearchSwitzerland; Stowers Institute for Medical ResearchUnited States

In the interests of transparency, eLife includes the editorial decision letter and accompanying author responses. A lightly edited version of the letter sent to the authors after peer review is shown, indicating the most substantive concerns; minor comments are not usually included.

Thank you for submitting your article "Actomyosin regulation by Eph receptor signaling couples boundary cell formation to border sharpness" for consideration by *eLife*. Your article has been reviewed by three peer reviewers, and the evaluation has been overseen by Marianne Bronner as the Senior and Reviewing Editor. The following individuals involved in review of your submission have agreed to reveal their identity: Filippo M Rijli (Reviewer #1); Robb Krumlauf (Reviewer #2).

The reviewers have discussed the reviews with one another and the Reviewing Editor has drafted this decision to help you prepare a revised submission.

Summary:

This interesting study by Cayuso et al. addresses ae long standing question of how border sharpening and boundary cell formation are coupled. By examining these events in the developing zebrafish hindbrain, they show that boundary cell markers are induced in EphA4-expressing cells and are up-regulated by forward signaling in rhombomeres 3 and 5. They show that myosin II phosphorylation downstream of EphA4 activation increases mechanical tension at segment borders, in turn inducing boundary cell markers via nuclear translocation of Taz.

Their work couples for the first time boundary sharpening by mechanical forces with the induction of boundary-specific genes, via EphA4 forward signaling. The findings are highly significant and novel. While the study is very well done, a few changes described below would improve the paper and render it ready for publication. The detailed reviews are included below for additional clarification.

Essential revisions:

1) The Taz phenotype in *epha4^-/-^* at 16hpf presented in Figure 6D is currently not very convincing. The authors' case would be strengthened by presenting the Taz phenotype in *epha4^-/-^* at 18hpf.

2) Genotyping data for CRISPants should be added as well as validation of the effects of the MO on protein levels should be added.

3) Regarding whether EphA4 forward signaling drives all the downstream actin rearrangement/Taz localization, the data suggests that this occurs via forward signaling, but are not conclusive. While adding data from a forward signaling mutant would be optimal, this is not essential but would ask that you soften this point by modifying text.

*Reviewer #1:*

This elegant study addresses one long standing and elusive key question concerning rhombomere (r) compartment formation in the developing hindbrain, namely how border sharpening and boundary cell formation are coupled. Jordi Cayuso and colleagues now beautifully identify the underlying mechanism in the hindbrain of the zebrafish embryo. They show that, in r3 and r5, boundary cell markers are induced in *epha4*-expressing cells and are up-regulated by forward signaling. Moreover, they demonstrate that myosin II phosphorylation downstream of *epha4* activation increases mechanical tension at segment borders, which in turn results in induction of boundary cell markers via nuclear translocation of Taz. Thus, Eph signaling couples border sharpening with boundary cell formation through mechanotransduction and Taz/Tead nuclear regulation of gene expression. These findings provide very important novel insights and will be of great interest to the readers of *eLife*.

A critical demonstration linking *epha4* signaling to regulation of border gene expression by Taz-mediated mechanotransduction is provided in Figure 6. However, the presentation of data in Figure 6D (*epha4^-/-^*) must be improved. First, the stage of Figure 6D is not mentioned on the panel nor in the figure legend. I assume is 16phf, as in Figure 6B. If so, then the distribution/placement of the yellow arrowheads when comparing Figure 6B and D makes difficult to appreciate the described reduction in nuclear Taz at the affected boundaries. Maybe using asterisks in addition to yellow arrowheads could help. Also, as it might be difficult to fully appreciate the defect in Taz localization at the 16hpf stage, the authors may add a quantification of the results as they did for *MO-mypt1* in Figure 6I. Nonetheless, it appears that the nuclear localisation of Taz at boundaries at 16hpf is still not fully refined and may suffer from a certain variability, even among wild type embryos. In contrast, Taz expression pattern at boundaries is very clear at 18hpf, as in Figure 6C. I believe therefore the analysis would very much benefit from presenting the Taz phenotype in *epha4^-/-^* at 18hpf, which would further strengthen the authors' case. Lastly, the stage in Figure 6E should be mentioned as well – i.e. 18hpf, and Figure 6G, G', H, H' are not 14hpf but 18hpf.

*Reviewer #2:*

This is a very interesting and comprehensive study which investigates the fundamental question of whether morphogenetic processes, associated with sharpening of inter-rhombomeric boundaries in the zebrafish hindbrain, are coupled with cell fate determination events that create unique boundary cells. They utilize gene editing to create an allelic series of EphA4 genes to clearly demonstrate that forward signaling from EphA4 is required to establish boundary cell identities and this process involves dual inputs of kinase and PDZ signaling. These experiments indicate that there is a tight coupling of boundary sharpening and formation of boundary cell identities regulated by EphA4.

While published studies from these authors and other have previously analyzed events and processes in boundary sharpening this study provides novel insight into mechanisms of cell fate determination modulated by EphA4 signaling that govern formation of boundary cell identity. They show that mechanical tension gated by phosphorylation of MLC directly impacts the regulation of boundary cell identity. This involves actomyosin dependent nuclear localization of Taz that regulates boundary cell. identity markers.

This a very strong paper because the authors elegantly show through genetic manipulations and cellular analyses precisely how the opposing domains of EphA4 and Efnb3 trigger a series of events that coordinates morphogenetic process of cell sharpening directly to cell differentiation. This is important because it provides a means for regulating the neural progenitor stem population at rhombomere boundaries. These findings have significant relevance beyond the formation hindbrain boundaries as this type of signaling is important in other contexts where cell sharpening and differentiation programs appear to correlate with EphA4 signaling.

The figures and data supporting the conclusions are very clear and beautifully presented. I believe this is very high quality work, with novel insight and broad general interest to cell and developmental biologists. My only comment is that I would have liked to have seen a summary/model that illustrates the steps in these processes. I believe it would help tie this together and reinforce the findings with the reader.

*Reviewer #3:*

In this manuscript, Cayuso et al. report that forward activation of EphA4 via the kinase and PDZ domains leads to actomyosin phosphorylation and actin contraction at segment borders, which in turn increases nuclear localization of Taz that induces the expression of boundary markers by interacting with Tead1a. This is a very interesting work, which couples for the first time boundary sharpening by mechanical forces with the induction of boundary-specific genes, via EphA4 forward signaling. These findings are significant and novel. Therefore, in principal I fully support its publication in *eLife*. Yet, there are some weaknesses in the data that should be addressed prior to its acceptance.

- The study uses many types of KO and KD techniques to eliminate the expression of multiple genes. However, no demonstration of the genotypes of the Crisp-Cas lines/Crispr-Cas transiently injected embryos as well as the loss of protein expression in the MO-injected embryos is provided to support the genetic and/or expression loss of the desired sequence/protein. These validations are necessary in the manuscript.

- A general EphA4 KO led to loss of pMLC in r2/r3, r3/r4 r5/r6 borders, as well as prevented the nuclear localization of Taz. However, there is no confirmation that these effects occur via the forward signaling only. Although the authors showed that the border expression of *rfng* and *wnt* is induced by the forward signaling of EphA4, these data are not sufficient to conclude that the entire pathway upstream to *rfng* expression is mainly mediated through the forward signaling. Only by using of the different EphA4 mutants in additional levels the pathway the authors can argue that the mechanical stabilization and Taz nuclear localization in the borders is mediated via the forward signaling.

- The authors don't show the pattern of the actin cables/pMLC in the *mypt1* KO. This is necessary in order to fully support the suggested model. Moreover, also in the rescues it would be nice to show not only the *rfng* expression rescue but the actual rescue if the actin cables/pMLC, as these are the actual readouts of the mechanical forces induced by EphA4.

- Taz KO/KD leads to loss of *rfng* in all boundaries, even in those not affected by *epha4* KO. Moreover, Tead1a KO leads to loss of *rfng* in a much milder way than Taz KO. These findings have to be explained in view of the suggested model of activation.

- The quality of the data in Figure 6 is poor. The nuclear localization data of Taz upon the different manipulations are hard to evaluate by the DAPI+ immunostaining. This is a very significant part of the study, and the authors have to present the cytoplasm-to-nuclear localization in a better way.

# Reply: Author response

**DOI:** 10.7554/eLife.49696.018

Essential revisions:

1) The Taz phenotype in epha4^-/-^ at 16hpf presented in Figure 6D is currently not very convincing. The authors' case would be strengthened by presenting the Taz phenotype in epha4^-/-^ at 18hpf.

We have repeated the immunostainings at 18 hpf and present better data. We now also show examples of nuclear localisation in different boundaries in Figure 6—figure supplement 1. We have clarified that there is an increase in Taz levels at boundaries, consistent with evidence that the actin cytoskeleton can regulate the stability as well as nuclear localisation of Taz (subsection “Myosin regulation downstream of EphA4 regulates Taz localization”, first paragraph). Not all boundary cells have nuclear Taz, which may reflect dynamic nuclear localisation.

2) Genotyping data for CRISPants should be added as well as validation of the effects of the MO on protein levels should be added.

We now present these data in Figure 2—figure supplement 1 and describe our and previous validations of MOs in Materials and methods (subsection “Generation of mutants”). All phenotypes from MO knockdowns have been validated by Crispr-mediated knockout. We discuss this in more detail in the response to reviewer 3.

3) Regarding whether EphA4 forward signaling drives all the downstream actin rearrangement/Taz localization, the data suggests that this occurs via forward signaling, but are not conclusive. While adding data from a forward signaling mutant would be optimal, this is not essential but would ask that you soften this point by modifying text.

This is a good point, as analysing Taz localisation in the different mutants would test whether it correlates with boundary cell formation. We have now analysed Taz localisation in the forward signaling mutants (truncated and kinase dead EphA4) and find that the same boundaries are disrupted as in the null mutant.

Reviewer #1:

[…] A critical demonstration linking epha4 signaling to regulation of border gene expression by Taz-mediated mechanotransduction is provided in Figure 6. However, the presentation of data in Figure 6D (epha4^-/-^) must be improved. First, the stage of Figure 6D is not mentioned on the panel nor in the figure legend. I assume is 16phf, as in Figure 6B. If so, then the distribution/placement of the yellow arrowheads when comparing Figure 6B and D makes difficult to appreciate the described reduction in nuclear Taz at the affected boundaries. Maybe using asterisks in addition to yellow arrowheads could help. Also, as it might be difficult to fully appreciate the defect in Taz localization at the 16hpf stage, the authors may add a quantification of the results as they did for MO-mypt1 in Figure 6I. Nonetheless, it appears that the nuclear localisation of Taz at boundaries at 16hpf is still not fully refined and may suffer from a certain variability, even among wild type embryos. In contrast, Taz expression pattern at boundaries is very clear at 18hpf, as in Figure 6C. I believe therefore the analysis would very much benefit from presenting the Taz phenotype in epha4^-/-^ at 18hpf, which would further strengthen the authors' case. Lastly, the stage in Figure 6E should be mentioned as well – i.e. 18hpf, and Figure 6G, G', H, H' are not 14hpf but 18hpf.

These are good points. We have repeated the immunostainings at 18 hpf and present better data. We now also show examples of nuclear localisation in different boundaries in Figure 6—figure supplement 1. We have clarified (subsection “Myosin regulation downstream of EphA4 regulates Taz localization”, first paragraph) that there is an increase in Taz levels at boundaries, consistent with evidence that the actin cytoskeleton can regulate the stability as well as nuclear localisation of Taz. Not all boundary cells have nuclear Taz, which may reflect dynamic nuclear localisation. We now mention the embryo stage for each panel on the figure and in the legend.

Reviewer #2:

[…] My only comment is that I would have liked to have seen a summary/model that illustrates the steps in these processes. I believe it would help tie this together and reinforce the findings with the reader.

Thank you for these helpful ideas. We now include a summary model as Figure 7. We tried to provide a broader context on possible relevance to other tissues in the subsection “Concluding Perspectives”, and we have now improved this section.

Reviewer #3:

[…] There are some weaknesses in the data that should be addressed prior to its acceptance.

- The study uses many types of KO and KD techniques to eliminate the expression of multiple genes. However, no demonstration of the genotypes of the Crisp-Cas lines/Crispr-Cas transiently injected embryos as well as the loss of protein expression in the MO-injected embryos is provided to support the genetic and/or expression loss of the desired sequence/protein. These validations are necessary in the manuscript.

We now include our data for the deletions in transient gene knockouts and the relevant sequence information for mutants (Figure 2—figure supplement 1). The MOs used have previously been described and validated by others – we now make this clearer in the Materials and methods (subsection “Generation of mutants”). For all MO-mediated knockdowns, we also carried out transient Crispr-mediated knockouts to ascertain whether this leads to the same phenotype. When possible, we validated the effect of MO on protein level. We show that the Taz MO (Hong et al., 2005) leads to a decreased level of protein expression (Figure 2—figure supplement 1). For the *mypt1* MO, there is to our knowledge no antibody available to detect the zebrafish protein. *mypt1* MO and mutant embryos were previously shown (Gutzman and Sive, 2010) to alter cell shape and to increase phosphorylation of MLC, which is a target of Mypt1. Likewise, we find that *mypt1* morphant and mutant embryos have the same phenotype (increased boundary marker expression). The *yap1* MO has been validated by Fukui et al. (2014) by rescue and by analysis of splicing, and also used by Cebola et al., Nat Cell Biol 17, 615-626, 2015; Agarwala et al., *eLife* e08201, 2015; Kozlovskaja-Gumbriene et al., *eLife* e21049, 2017.

- A general EphA4 KO led to loss of pMLC in r2/r3, r3/r4 r5/r6 borders, as well as prevented the nuclear localization of Taz. However, there is no confirmation that these effects occur via the forward signaling only. Although the authors showed that the border expression of rfng and wnt is induced by the forward signaling of EphA4, these data are not sufficient to conclude that the entire pathway upstream to rfng expression is mainly mediated through the forward signaling. Only by using of the different EphA4 mutants in additional levels the pathway the authors can argue that the mechanical stabilization and Taz nuclear localization in the borders is mediated via the forward signaling.

We have now analysed Taz localisation in the forward signaling mutants (truncated and kinase dead EphA4) and find that the same boundaries are disrupted as in the null mutant. As we have not analysed pMLC in the forward signaling mutants, we have softened our discussion of this point (subsection “EphA4 signaling and boundary cell formation”). A number of studies have shown that forward signaling is the main driver of segregation and border sharpening, and acts by increasing actomyosin tension (Canty et al., 2017; Fagotto et al., 2013; O’Neill et al., 2016; Rohani et al., 2011, 2014). However, we have shown in a cell culture model that reverse signaling leads to weaker repulsion that can contribute to cell segregation (Taylor et al., 2017; Wu et al., 2019). Nevertheless, if reverse signaling does lead to some increase in pMLC at boundaries, this is not sufficient to induce boundary gene expression in forward signaling mutants, even after amplifying tension by *mypt1* knockdown.

- The authors don't show the pattern of the actin cables/pMLC in the mypt1 KO. This is necessary in order to fully support the suggested model. Moreover, also in the rescues it would be nice to show not only the rfng expression rescue but the actual rescue if the actin cables/pMLC, as these are the actual readouts of the mechanical forces induced by EphA4.

We do not show the increase in MLC phosphorylation after *mypt1* knockdown as this had been shown by Gutzman and Sive (2010) by Western blot analysis and immunostaining of the hindbrain. They were not able to detect pMLC at hindbrain boundaries in wild type embryos, but did when pMLC increases in the *mypt1* mutant. Their Western blot analysis revealed a five-fold increase in pMLC after *mypt1* knockdown. By optimising the immunostaining we were able to detect pMLC at hindbrain boundaries in wild type embryos. This extends the previous findings of myosin and actin cables at boundaries, which Calzolari et al. (2014) showed are EphA4-dependent. We agree that it would be nice to show that *mypt1* knockdown increases pMLC at boundaries in the EphA4 mutant, but feel that our findings together with the published work give compelling evidence for the pathway from EphA4 –> pMLC –> Taz –> boundary gene expression.

- Taz KO/KD leads to loss of rfng in all boundaries, even in those not affected by epha4 KO. Moreover, Tead1a KO leads to loss of rfng in a much milder way than Taz KO. These findings have to be explained in view of the suggested model of activation.

We have clarified these points in the revised manuscript (subsection “*taz* and *tead1a* are required for boundary marker expression”). That Taz knockout affects all hindbrain boundaries is consistent with increased pMLC and Taz nuclear localisation at all segment borders. EphB4 and ephrinB2 are segmentally expressed and drive cell segregation, and likely contribute to pMLC and boundary marker expression at the r1/r2, r5/r6 and r6/r7 borders that are not affected in the EphA4 mutants (mentioned in the Introduction and Results). However, we have found that there is some redundancy at these borders, and that at least one further Eph-ephrin pair is segmentally expressed. We now mention (see the aforementioned subsection) that the milder phenotype of tead1a knockout may be due to partial redundancy with family members that have not yet been analysed.

- The quality of the data in Figure 6 is poor. The nuclear localization data of Taz upon the different manipulations are hard to evaluate by the DAPI+ immunostaining. This is a very significant part of the study, and the authors have to present the cytoplasm-to-nuclear localization in a better way.

We agree that the quality of the staining is not great, which unfortunately reflects that the Taz antibody is tricky to use. For example, Voltes et al. (2019) analysed later stages and detected increased Taz protein but not its nuclear localisation at boundaries (they obtained indirect evidence for pathway activation by using a Tead reporter gene). We tested different conditions and found that it is important to carry out antigen retrieval (now mentioned in the subsection “Immunohistochemistry and in situ hybridization”). We have carried out stainings for *epha4* null, truncated and kinase dead mutants and present the new data (revised Figure 6). We also clarified (subsection “Myosin regulation downstream of EphA4 regulates Taz localization”, first paragraph) that there is an increase in Taz levels at boundaries, consistent with evidence that the actin cytoskeleton can regulate the stability of Taz. Not all boundary cells have nuclear Taz, which may reflect dynamic nuclear localisation. We now show examples of nuclear localisation in different boundaries (Figure 6—figure supplement 1).
